# Krüppel-like transcription factors and control of pluripotency

**DOI:** 10.1186/1741-7007-8-125

**Published:** 2010-09-27

**Authors:** Pierre-Yves Bourillot, Pierre Savatier

**Affiliations:** 1 Inserm U846, 18 avenue Doyen Lépine, 69500 Bron, France; 2 Stem Cell and Brain Research Institute, 69500 Bron, France; 3 Université de Lyon, Université Lyon 1, 69003, Lyon, France

## Abstract

Recent papers have demonstrated a role for Krüppel-like transcription factors 2, 4 and 5 in the control of mouse embryonic stem cell pluripotency. However, it is not clear whether each factor has a unique role or whether they are functionally redundant. A paper by Parisi and colleagues in *BMC Biology *now sheds light on the mechanism by which Klf5 regulates pluripotency.

See research article http://www.biomedcentral.com/1741-7007/8/128

## Klfs induce and maintain pluripotency

Krüppel-like factors (Klfs) are evolutionarily conserved zinc finger-containing transcription factors implicated in many biological processes, including proliferation, apoptosis, differentiation and development. Recently, Klfs received renewed attention following the demonstration that somatic cells can be reprogrammed into induced pluripotent stem (iPS) cells using a cocktail of transcription factors that includes Klf4. More recently, a large body of evidence has accumulated that expression of *Klf2*, *Klf4 *and *Klf5 *genes is associated with pluripotency control. They are highly expressed in mouse embryonic stem cells (ESCs) and this expression drops dramatically after induction of differentiation by withdrawal of leukemia inhibitory factor (LIF) or suspension culture [1]. Functional inactivation of any one of these genes by RNA interference in ESCs induces spontaneous differentiation [1-3], whereas overexpression harnesses self-renewal and delays differentiation induced by the formation of embryoid bodies [2-4]. *Klf5^-/- ^*embryos fail to develop beyond the blastocyst stage *in vivo *or to produce ESC lines *in vitro *[5], a finding consistent with Klf5 controlling the pluripotency of the epiblast, the embryonic tissue from which ESCs originate. One question raised by these recent findings is whether Klf2, Klf4 and Klf5 have redundant functions in pluripotency, or whether each factor plays a unique role in the maintenance of the undifferentiated state of ESCs. The article now published by Parisi *et al*. in *BMC Biology *[6] compares the Klf5 regulon with those of Klf2 and Klf4 and concludes that Klf5 regulates the expression of a unique set of genes that distinguishes it from other Klf members. These findings support the notion that each Klf member might play a specific role in the maintenance of the pluripotent state.

## Klf2, Klf4 and Klf5 play contrasting roles in pluripotency

Several papers recently reported that ESC differentiation induced by *Klf2/Klf4/Klf5 *triple knockdown, homozygous disruption of *Klf5*, or withdrawal of the cytokine LIF-which down-regulates *Klf *gene expression-could be rescued by overexpressing any one of the three *Klf *genes [2,3,7]. This observation suggests that *Klf2*, *Klf4 *and *Klf5 *exert redundant effects on the control of pluripotency. However, a closer look at the yield and the phenotype of Klf-rescued cells suggests that things are not that simple. A hierarchical relationship in the ability of Klfs to support ESC self-renewal in the absence of LIF was reported, with Klf2 being most potent, Klf4 being intermediate, and Klf5 being least potent [3]. This finding corroborates the earlier observation that Klf2 and Klf4 are far more efficient at reprogramming somatic cells into iPS cells than Klf5 [8]. Moreover, in comparison with wild-type ESCs propagated in the presence of LIF, *Klf5 *knockout ESCs exhibit a longer G1 phase when rescued with Klf4, and a shorter G1 when rescued with Klf5 [5]. This is in agreement with the observation made in somatic cells that Klf4 delays and Klf5 accelerates the G1/S transition by regulating the expression of cyclins, cyclin-dependent kinases (Cdk) and Cdk inhibitors. Last, but not least, knockdown of Klf4 biases ESC differentiation towards extraembryonic endoderm, whereas knockdown of Klf5 biases it towards mesoderm [1]. This observation strongly suggests that Klf4 and Klf5 inhibit two mutually exclusive differentiation programmes, and that both factors are necessary to maintain ES cells in a fully undifferentiated state. Whether and how the opposing roles of Klf4 and Klf5 in cell cycle regulation and inhibition of endoderm versus mesoderm differentiation are causally related is an issue that needs to be explored.

## Klf2, Klf4 and Klf5 are closely connected to the core pluripotency network

**Figure 1:**
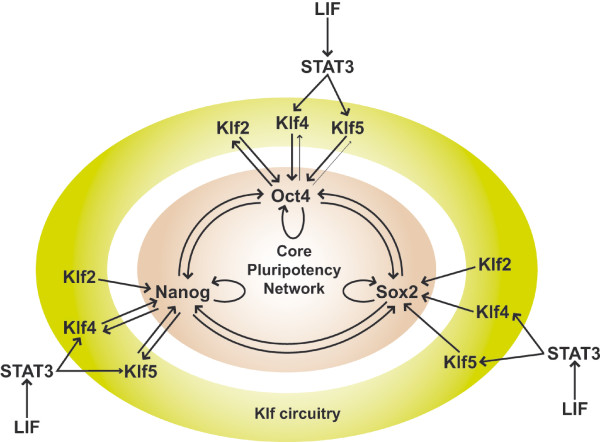
**Core transcriptional regulatory circuitry in pluripotent mouse ESCs**.

Klfs have also been implicated in the regulation of an autoregulatory network, known as the core pluripotency network, that plays a key role in ESC self-renewal. This network comprises the homeodomain transcription factors Oct4 (also known as Pou5f1) and Nanog, and the HMG-box transcription factor Sox2. The promoters of each of these genes contain binding sites for all three transcription factors and disruption of any of the three genes compromises pluripotency. Klfs and the Oct4/Sox2/Nanog network are strongly interconnected since (*i*) Klf2, Klf4 and Klf5 activate the expression of *Nanog*, *Sox2*, and *Oct4 *[7], (*ii*) *Klf2 *is activated by Oct4 [3], and (*iii*) *Klf4 *and *Klf5 *are activated by Nanog [1]. Thus, Klf2, Klf4, Klf5, Oct4, Sox2 and Nanog form a molecular circuitry essential to ESC self-renewal [3]. Furthermore, *Klf4 *and *Klf5 *- but not *Klf2 *- are regulated by the Signal transduction and activator of transcription (STAT)-3 following activation by LIF in mouse ESCs. This regulation makes Klf4 and Klf5 the missing link that connects extrinsic regulators to the core pluripotency network [1,9] (Figure 1). Significantly, following induction of differentiation by suspension culture or withdrawal of LIF, expression of *Klf4 *and *Klf5 *is downregulated very early, whereas expression of *Klf2 *is downregulated later [1,10]. This indicates a progressive deconstruction of the molecular circuitry that controls pluripotency during ESC differentiation.

## Convergent and divergent Klf2, Klf4 and Klf5 regulons

To get some insight into the mechanisms by which Klf5 controls ESC pluripotency, Parisi and colleagues [6] have explored Klf5-specific targets by matching microarray data from control and Klf5 knock-down cells with chromatin immunoprecipitation coupled high-throughput sequencing (ChIP-seq). They have thus identified 161 primary targets of Klf5, of which eight (out of 23 tested) were shown by RNA interference to contribute to maintenance of the undifferentiated state. Surprisingly, only 10% of 53 tested Klf5 targets are also regulated by both Klf2 and Klf4, and none of the genes encoding the core pluripotency network (*Oct4*, *Sox2*, and *Nanog*) appear to be among the Klf5 targets. Most likely, this is explained by a functional redundancy among Klf2, Klf4 and Klf5 in activating components of the core pluripotency network. Furthermore, 90% of the 53 tested Klf5 targets are not regulated by Klf4, a finding consistent with an earlier report by Jiang and colleagues [7] showing that 60% of the Klf5 targets lack a binding site for Klf4. Among the eight genes identified by Parisi and colleagues that contribute to the maintenance of the undifferentiated state, *Igfbp3*, *Niban *and *Perp *are three Klf5-specific targets. Interestingly, mining their data with those of Jiang and colleagues [7] led us to identify two more genes, *Bcam *and *Hck*, that are Klf4/Klf5-specific targets (that is, not regulated by Klf2). Similarly, we have identified *Foxd3*, a Klf4-specific target (that is, not regulated by Klf5), suppression of which impairs self-renewal (data not shown).

Collectively, these data shed light on a possible mechanism of Klf action in ESC self-renewal. On the one hand, Klf2, and Klf4 and Klf5 co-regulate the expression of master regulators of pluripotency, including *Oct4*, *Sox2 *and *Nanog*. Inactivation of all three *Klfs *is required to inactivate the core pluripotency network and trigger extensive differentiation. On the other hand, each Klf member regulates the expression of specific targets, thereby further promoting self-renewal via ancillary factors. Inactivation of individual *Klf *genes is thus detrimental to self-renewal but is not sufficient to trigger the massive differentiation observed following triple inactivation. However, it is conceivable that inactivation of a single *Klf *gene drives ESC into a new metastable state characterized by altered proliferation and differentiation features. In the future, a thorough analysis of the *Klf2*, *Klf4*, and *Klf5 *regulons associated with functional genomics will help understand how these three factors regulate the balance between self-renewal and differentiation into each one of the embryonic lineages.
